# Human Amnion Epithelial Cell Therapy for Chronic Liver Disease

**DOI:** 10.1155/2019/8106482

**Published:** 2019-08-07

**Authors:** Neil Andrewartha, George Yeoh

**Affiliations:** ^1^Centre for Medical Research, Harry Perkins Institute of Medical Research, QEII Medical Centre, Nedlands, Western Australia 6009, Australia; ^2^School of Molecular Sciences, The University of Western Australia, Crawley, Western Australia 6009, Australia; ^3^Centre for Cell Therapy and Regenerative Medicine, School of Biomedical Sciences, The University of Western Australia, Crawley, Western Australia 6009, Australia

## Abstract

Liver fibrosis is a common consequence of chronic liver disease. Over time, liver fibrosis can develop into liver cirrhosis. Current therapies for liver fibrosis are limited, and liver transplant is the only curative therapy for patients who progress to end-stage disease. A potential approach to treat chronic liver disease with increasing interest is cell-based therapy. Among the multiple cell types which have been proposed for therapeutic uses, human amnion epithelial cells and amniotic fluid-derived mesenchymal cells are promising. These cells are highly abundant, and their use poses no ethical concern. Furthermore, they exert potent anti-inflammatory and antifibrotic effects in animal models of liver injury. This review highlights the therapeutic characteristics and discusses how human amnion epithelial cells can be utilised as a therapeutic tool for chronic liver disease.

## 1. Introduction

Chronic liver disease (CLD) results in the development of chronic hepatic wound healing, characterised by persistent liver inflammation and the accumulation of extracellular matrix proteins (ECM), collectively described as fibrosis [1, 2]. Crucial to the pathogenesis of liver fibrosis are hepatic stellate cells (HSCs) and macrophages [3, 4]. HSCs propagate fibrosis by secreting ECM proteins and profibrotic factors while macrophages, consisting of resident Kupffer cells and those derived from infiltrating monocytes, perform a diversity of functions during hepatic wound healing [5, 6]. These include secreting a myriad of proinflammatory and profibrotic factors, recruiting other immune cell populations and clearing cell debris by phagocytosis. These activities perpetuate fibrosis which can develop into liver cirrhosis.

Cirrhosis is associated with reduced liver function and an increased risk of developing liver cancers. Currently, a liver transplant is the only cure for patients who progress to end-stage liver cirrhosis. However, transplantation is complicated by low organ availability, high cost, and long-term immunosuppression [7]. Consequently, it is imperative that a novel antifibrotic therapy for CLD be developed.

Research has highlighted the potential of stem cell-based therapies for CLD [8, 9]. For instance, mesenchymal embryonic and induced pluripotent stem cells have been recognised as possessing therapeutic properties relevant to CLD. However, the clinical realisation of these candidates is hindered by safety, cost, availability, and ethical considerations [9]. These issues have led scientists to seek alternative cell sources, of which perinatal stem cells are one of the most promising.

Perinatal stem cells are derived from extraembryonic tissues, such as the foetal membrane and umbilical cord [10, 11]. They share a unique ontogenetic relationship to the developing foetus, with some even arising prior to gastrulation. Their unique origin is thought to be the reason why perinatal cells combine the therapeutic qualities of adult stem cells, such as mesenchymal stem cells, with the differentiation potential of embryonic stem cells [12, 13]. Additionally, perinatal stem cells are immune privileged and genetically stable, meaning they do elicit an inflammatory immune response or form teratomas following transplantation in animal models [12, 14]. Finally, perinatal stem cells are isolated in abundance from material that is normally discarded after birth, so their use poses less ethical concern. Combined, these advantages make a strong case that perinatal stem cells are more practical for clinical use compared to other cell therapy candidates.

Over the decades, multiple cell types have been isolated from extraembryonic material, including the foetal membrane, umbilical cord, and amniotic fluid. Of these sources, one of the most extensively investigated is the amniotic component of the foetal membrane [15, 16]. The amniotic membrane is currently used in clinical ophthalmology and skin grafting due to its ability to reduce fibrosis and promote tissue repair [17–20]. It contains an epithelial and a mesenchymal cell population which, have been isolated and investigated as a therapeutic tool [11, 21, 22]. Both cell types demonstrate similar therapeutic properties; however, only the epithelial population can be isolated in a clinically compliant manner at numbers sufficient for clinical use [23]. As a result, the therapeutic properties of human amnion epithelial cells (hAECs) have been explored in animal models of the liver, lung, cardiac, epidermal, and neurological [22, 24–31]. These preclinical studies show that systemic infusion of hAECs attenuates inflammation and reduces fibrosis suggesting that they may be able to ameliorate chronic hepatic wound healing in patients with CLD.

## 2. Human Amnion Epithelial Cells Modulate Chronic Wound Healing

**Figure 1:**
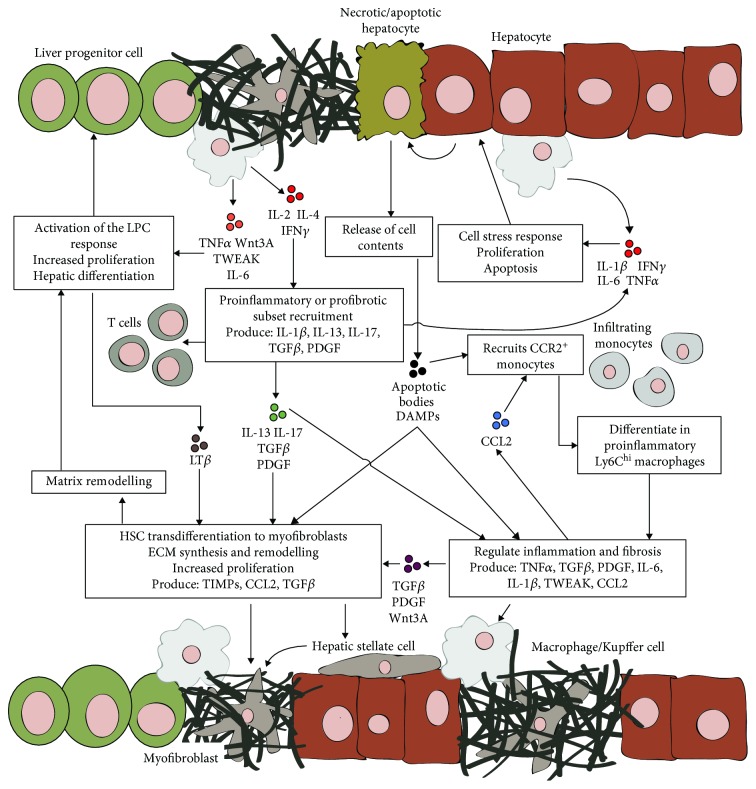
Overview of chronic hepatic wound healing and fibrosis. Necrotic and/or apoptotic hepatocytes release their cell content stimulating monocyte infiltration and hepatic stellate cell activation. Upon infiltration, monocytes differentiate into proinflammatory Ly6C^hi^ macrophages which, along with resident Kupffer cells, release a myriad of proinflammatory and profibrotic cytokines. These factors promote additional inflammatory cell recruitment, regulate tissue repair, and activate hepatic stellate cells. Activated stellate cells transdifferentiate into ECM-producing myofibroblasts. Myofibroblasts also augment ECM remodelling by producing TIMPs and regulate tissue repair by secreting inflammatory and profibrotic factors. As a consequence of liver inflammation, T cells are recruited which further promote inflammation and/or stellate cell activation through cytokine production. Finally, persistent hepatic inflammation, ECM remodelling, and hepatocyte injury activate the liver progenitor cell compartment. Liver progenitor cells proliferate and differentiate into hepatocytes to assist liver regeneration during CLD. CLD perpetuates this wound healing response resulting in persistent liver inflammation and the development of fibrosis.

Generally, chronic wound healing involves a highly complex and dynamic interplay between injured parenchymal cells, myofibroblasts, inflammatory cells, and tissue-specific stem/progenitor cells (Figure 1) [32, 33]. It has been suggested that due to this complexity, a multifactorial approach is needed to slow or reverse the progression of tissue fibrosis [34, 35]. Herein lies the value of hAECs; as studies show they suppress and modulate multiple aspects of chronic wound healing. Specifically, hAECs have been reported to (i) attenuate myofibroblast activation [24, 36, 37], (ii) suppress monocyte/macrophage recruitment [36, 38, 39], (iii) promote macrophage polarisation toward a reparative phenotype [36, 40, 41], and (iv) induce regulatory T-cell differentiation [42]. These effects are largely mediated by the paracrine factors secreted by hAECs [24, 36, 37, 43] as demonstrated in studies demonstrating that the benefits of hAEC therapy occur independently of cell engraftment [37, 44]. Furthermore, hAEC-conditioned medium is able to reduce liver fibrosis in a mouse model of chronic liver injury [24, 45]. Accordingly, identifying the trophic factors secreted by hAECs and how they affect chronic wound healing is an active area of research.

## 3. hAECs Inhibit Myofibroblast Activation

A hallmark of tissue fibrosis is the accumulation of ECM-producing myofibroblasts [1, 46]. Myofibroblasts are a crucial component of tissue repair as they secrete ECM proteins, regulate ECM remodelling, and produce inflammatory and fibrotic cytokines and chemokines [3]. Typically, these cells are cleared once injury and inflammation subside; however, during CLD, persistent inflammation perpetuates myofibroblast activation resulting in the progressive accumulation of ECM in the liver [47].

Myofibroblasts can be derived from a variety of precursor cells type; however, liver myofibroblasts are almost exclusively derived from hepatic stellate cells (HSCs) [4]. These normally quiescent perisinusoidal cells found in the Space of Disse activate during liver injury and transdifferentiate into liver myofibroblasts. To this end, suppressing HSC activation and promoting the clearance of myofibroblasts is a major goal in the development of antifibrotic therapies.

Preclinical studies in animal models of the liver, lung, and skin fibrosis report that hAEC therapy reduces the number of myofibroblasts within injured tissue [24, 31, 36, 40, 48]. Furthermore, in the murine models of chronic carbon tetrachloride- (CCl_4_-) induced liver injury and bleomycin-induced lung injury, hAEC therapies are reported to reduce the levels of profibrotic factors, namely, transforming growth factor-*β* (TGF*β*) and platelet-derived growth factor (PDGF) [24, 36, 40]. TGF*β* stimulates HSC activation, maintains myofibroblast survival, and promotes ECM synthesis. Furthermore, TGF*β* reduces the activity of matrix-degrading matrix metalloproteinases (MMPs) by upregulating the expression of tissue inhibitor of metalloproteinases (TIMPs) by myofibroblasts [49]. PDGF is a potent mitogen for myofibroblasts and is upregulated by liver injury [50]. Thus, by downregulating the production of these factors, hAEC therapy attenuates HSC activation and ECM synthesis.

In addition to reducing TGF*β* and PDGF activity, hAECs directly suppress the fibrotic activity of HSCs and myofibroblasts through paracrine signalling. For instance, Hodge et al. demonstrated that when cultured in hAEC-conditioned medium, HSCs adopt an antifibrotic phenotype characterised by a reduction in proliferation, activation, and ECM production [51]. Furthermore, Zhao et al. demonstrated that hAECs block TGF*β* signalling in myofibroblasts by secreting soluble human leukocyte antigen G5 [48]. Other antifibrotic factors inducing prostaglandin E2, bone morphogenetic protein-7, and interleukin-10 are also secreted by hAECs [51]. Overall, these studies indicate that hAECs attenuate the profibrotic activity of HSCs and myofibroblasts, through both direct and indirect mechanisms.

## 4. hAECs Modulate Macrophage Recruitment

CLD is closely associated with the enrichment of liver macrophages [52]. Macrophages promote inflammation and fibrosis by secreting a host of cytokines including TGF*β*, PDGF, interleukin- (IL-) 1, IL-6, tumor necrosis factor alpha (TNF*α*), and TNF-related weak inducer of apoptosis (TWEAK) [5, 53]. These factors perpetuate inflammation, stimulate and maintain myofibroblast activation, and induce immune-mediated tissue injury [54–57]. Consequently, macrophages have become an attractive target for antifibrotic therapies.

The liver houses a specialised macrophage population known as Kupffer cells; however, following injury, the liver's macrophage population expands dramatically through monocyte recruitment. These monocytes differentiate into monocyte-derived macrophages (MDMs) upon infiltration into the injured liver [58]. Research suggests that Kupffer cells are imperative for maintaining liver homeostasis and the early response to liver injury [59]. In comparison, MDMs are crucial for inflammation and tissue repair following liver injury [60]. In fact, during liver repair, the number of Kupffer cells decreases while there is a substantial increase in the number of MDMs [61, 62]. Furthermore, the development of inflammation and fibrosis in other tissues such as the lung and kidney is associated with the recruitment of MDMs [63]. Therefore, suppressing MDM recruitment could alleviate liver inflammation and fibrosis.

The benefits of hAEC therapy are associated with reduced macrophage recruitment. Studies using bleomycin-induced lung injury and liver injury caused by CCl_4_ or a high fat diet show a reduction in macrophage numbers when hAECs are infused systemically [36, 38–40, 45]. Accordingly, the expression levels of their associated factors including TGF*β*, PDGF, IL-1, IL-6, and TNF*α* are reduced [22, 36, 38–40]. This outcome may be related to a decrease in the expression levels of the chemokine CCL2 [36, 39]. CCL2 recruits CCR2-expressing monocytes and is considered a key mediator of MDM recruitment following injury [58, 64]. In fact, attenuation of CCL2-CCR2 signalling either by genetic or by pharmacological means reduces MDM recruitment and hepatic fibrosis in murine models [58, 65]. Furthermore, both serum levels and liver expression of CCL2 are reported to correlate with the severity of CLD [66]. Hence, suppression of CCL2/CCR2 MDM recruitment by hAECs may be of therapeutic value to patients with CLD.

## 5. hAECs Alter Macrophage Polarisation

Animal models of macrophage depletion have shown that macrophages can both promote and resolve tissue fibrosis. For example, Duffield et al., using the CD11b-DTR transgenic mouse, established that macrophage depletion during liver injury and inflammation prevents the development of liver fibrosis. Conversely, depletion during recovery attenuates the degradation of matrix proteins [67]. These contrasting roles highlight the plasticity of macrophages. Macrophages will adapt their phenotype in response to signals from their microenvironment. In general, these phenotypes are classified as classical (M1) or alternatively (M2) activated [68].

**Figure 2:**
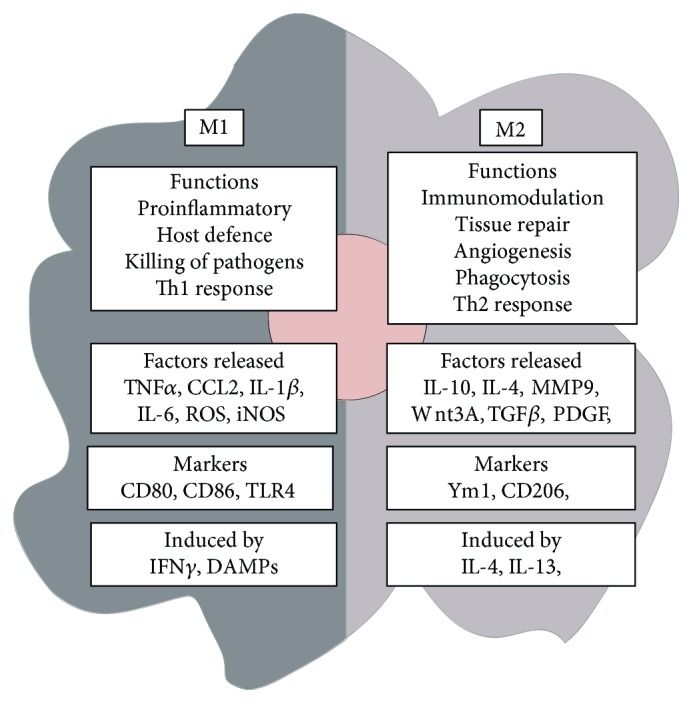
M1/M2 paradigm of macrophage polarisation. The M1/M2 paradigm describes alternative states of macrophage polarisation with each exerting different functions in inflammation and fibrosis. The activation state adopted by macrophages is dependent on signalling molecules from their microenvironment.

M1 macrophages are predominantly proinflammatory, releasing cytokines such as TNF*α*, IL-1*β*, and IL-6 [68, 69]. In contrast, M2 macrophages are associated with immunomodulation and tissue repair [68, 69]. M2 macrophages display a higher capacity for phagocytosis and secrete factors including IL-10, TGF*β*, and MMPs [68, 70] (Figure 2). It is important to highlight that the M1/M2 classification system oversimplifies the heterogeneity of macrophage phenotypes in disease conditions. Macrophages often express M1 and M2 activation markers simultaneously, so rather than two distinct subpopulations, the M1/M2 paradigm signifies a spectrum of activation states. Nonetheless, the transition from predominantly M1 to M2 during wound healing is associated with the resolution of inflammation and initiation of tissue repair [68, 71]. This transition becomes dysregulated during CLD resulting in chronic inflammation, dysfunctional wound healing, and fibrosis. Therefore, modulating macrophage polarisation to limit chronic inflammation and fibrosis in patients with CLD is an attractive strategy.

Evidence suggests that hAEC therapy augments macrophage polarisation toward an M2 phenotype [24, 36–38, 72]. The expression of M2-associated markers including CD206, IL-10, and MMP9 is increased by hAEC therapy in murine models of CCl_4_-induced liver and bleomycin-induced lung injuries [24, 36, 40, 41]. IL-10 is a potent anti-inflammatory cytokine known to suppress monocyte infiltration, synthesis of proinflammatory mediators, and collagen synthesis by myofibroblasts [73, 74]. In fact, a clinical trial of IL-10 in patients with chronic hepatitis C reported a reduction in fibrosis and improvements in liver histology and function [75]. Similarly, MMP9, which breaks down collagen, is reported to exert an antifibrotic effect in murine models of chronic liver injury [6, 76]. It is important to highlight that hAEC therapy does not promote the profibrotic functions of M2 macrophages through their secretion of TGF*β* and PDGF. In fact, the M2 functions that are induced by hAEC therapy are associated with immunosuppression and breakdown of ECM.

**Figure 3:**
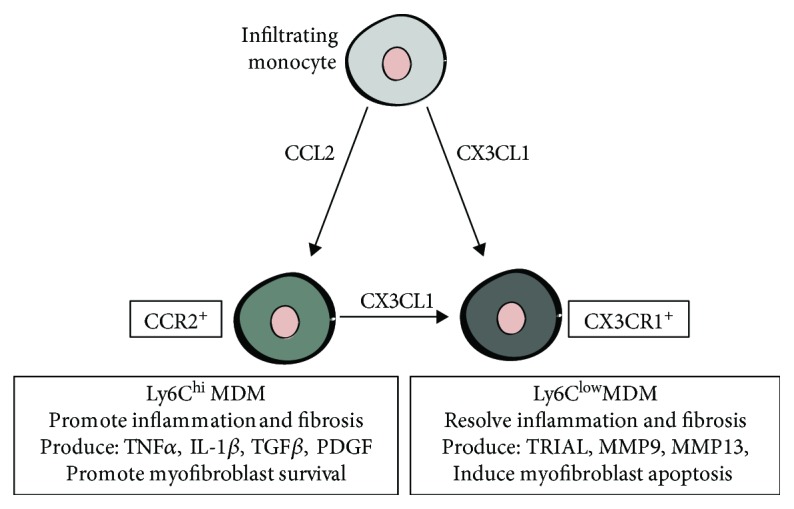
Differential expression of Ly6C distinguishes MDMs with opposing functions in tissue repair. During liver repair, monocytes recruited by the CCL2/CCR2 axis differentiate into profibrotic Ly6C^hi^-expressing MDMs. In contrast, monocytes recruited by the CX3CL1/CX3CR1 axis give rise to Ly6C^low^ MDMs. This subpopulation promotes the resolution of tissue repair and regression of fibrosis. CX3CL1 can induce a phenotypic switch of Ly6C^hi^ MDMs to a Ly6C^low^ phenotype.

It is likely that soluble factors secreted by hAECs play an important role in their modulation of macrophages. For instance, Tan et al. demonstrated that hAECs secrete lipoxin A4 which promotes macrophage phagocytosis in culture [43]. Interestingly, lipoxin A4 has been shown to reduce inflammation and fibrosis by promoting M2 polarisation in a mouse model of obesity-induced hepatic injury [77]. Similarly, the production of soluble human leukocyte antigen G5 by hAECs may induce M2 polarisation [78]. Furthermore, hAECs are reported to produce the chemokine CX3CL1 which induces MDMs to differentiate into the Ly6C^low^ phenotype [79, 80] (Figure 3). This macrophage subpopulation protects against liver fibrosis and is critical for its resolution [61]. However, while hAEC therapy does increase hepatic expression of CX3CL1 during chronic CCl_4_-induced liver injury, it is currently unknown whether this translates to an increase in Ly6C^low^ MDMs in this context [36]. Nevertheless, these studies suggest that multiple soluble factors secreted by hAECs may promote a macrophage phenotype that resolves liver fibrosis.

## 6. hAECs Promote Regulatory T Cells

**Figure 4:**
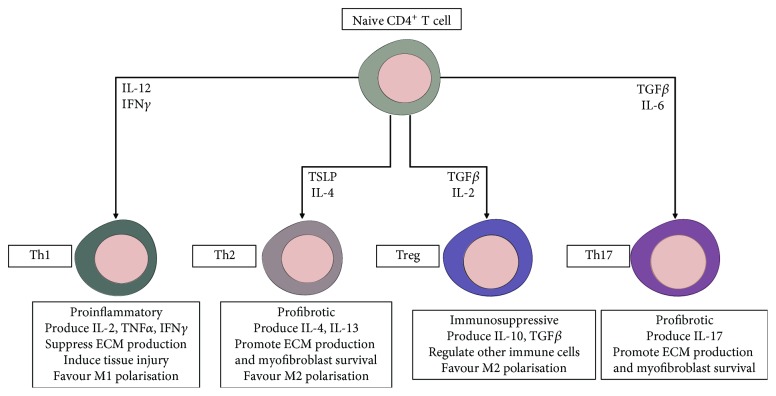
Functions of CD4^+^ T-cell subsets in liver fibrosis. The differentiation of naive CD4^+^ T cells into distinct functional subtypes is driven by factors produced by injured parenchymal cells and other inflammatory cells, namely, macrophages. Tregs suppress other T-cell subsets and limit the magnitude of inflammation and fibrosis during tissue repair.

Regulatory T cells (Tregs) are important regulators of inflammation and fibrosis. This subset of CD4^+^ T helper cells suppresses the immune response by secreting immunosuppressive factors including IL-10 and TGF*β*. Furthermore, Tregs promote M2 macrophage polarisation and counteract the activity of other CD4^+^ T-cell subsets (Figure 4). An imbalance between T-cell subsets is an important factor in the progression of CLD. For example, an excessive Th1 and/or Th17 response is associated with increased severity of chronic hepatitis B [81]. In fact, Gu et al. demonstrated that rapamycin ameliorates liver inflammation and fibrosis by upregulating Tregs and downregulating Th17 cells in murine models [82]. Therefore, promoting Treg maturation has the potential to reduce liver fibrosis in patients with CLD.

The induction and expansion of Tregs is a crucial component of hAEC therapy. hAECs increase the number of Tregs in the lung following bleomycin-induced injury [42]. Additionally, hAEC therapy prevents bleomycin-induced lung damage in Rag1^−/−^ mice, only when an adoptive transfer of either Tregs or naive T cells is coadministered [42]. Importantly, research suggests that enhancing Treg activity ameliorates inflammation and fibrosis in a mouse model of chronic liver injury [83]. Conversely, Treg depletion during chronic liver injury exacerbates liver inflammation and fibrosis [84]. Therefore, the upregulation of Treg activity by hAECs may achieve beneficial outcomes in patients with CLD.

## 7. hAECs May Support Endogenous Liver Regeneration

Most studies investigating hAEC-based therapies for liver pathologies focus on the inflammatory and fibrotic aspects. Currently, it is unknown whether hAEC therapy enhances hepatocyte regeneration. Regardless, as hAEC therapy improves markers of tissue function in models of chronic liver and lung injury, it is likely that tissue regeneration is occurring [24, 36, 37].

**Figure 5:**
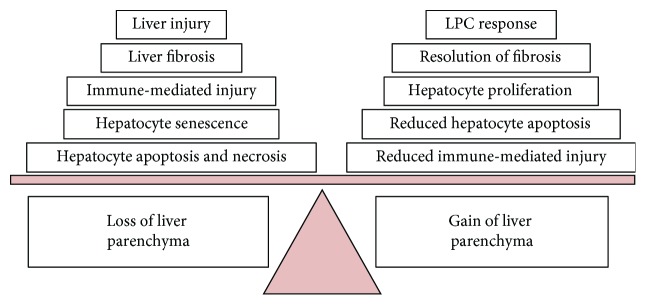
Mechanisms that contribute to the loss or gain of liver parenchyma during CLD. Liver repair following injury involves a balancing act between mechanisms that result in either loss or gain of the liver parenchyma. Accordingly, the progression of CLD can be framed as liver repair that favours parenchyma loss over gain. Flipping this balance in favour of parenchyma gain is the ultimate goal of regenerative therapies for CLD.

hAEC therapy may support liver regeneration through multiple mechanisms. Firstly, hAECs secrete growth factors such as EFG and IGF2 which are known to induce hepatocyte proliferation [24, 85, 86]. Secondly, since the fibrotic matrix is a major inhibitor of hepatocyte proliferation [87], the antifibrotic properties of hAECs, particularly their ability to suppress HSC activation, should assist liver regeneration. In addition, the ability of hAECs to upregulate MMP and downregulate TIMP expression may encourage regeneration by promoting ECM degradation [36, 51, 87]. Finally, hAEC therapy reduces hepatocyte apoptosis during CCl_4_-indcued liver injury [22, 36]. This is likely mediated by their immunomodulatory effects, as factors including TNF*α*, IL-1*β*, and TGF*β* are known to stimulate hepatocyte apoptosis [88, 89]. Excessive hepatocyte apoptosis is a common feature of CLD and directly contributes to the progressive loss of liver parenchyma [88, 89]. Therefore, by tempering hepatocyte apoptosis, hAECs may be able to mitigate the progressive loss of the liver parenchyma in patients with CLD and promote regeneration. Collectively, these studies suggest that through a combination of both direct and indirect mechanisms, hAEC therapy may augment liver repair to favour hepatocyte regeneration (Figure 5).

hAEC therapy may also promote hepatocyte regeneration by stimulating the proliferation and differentiation of liver progenitor cells (LPCs). LPCs are a population of bipotential epithelial cells that reside in the canals of Hering, located within the biliary tree [90, 91]. LPCs are rare in a healthy liver; however, during persistent liver injury, especially where there is chronic inflammation, fibrosis, and hepatocyte senescence, LPCs emerge from the bile canaliculi, proliferate, and differentiate into hepatocytes or cholangiocytes. This response is frequently referred to as the ductular reaction and is observed in patients with viral hepatitis, alcoholic liver disease, and fatty liver disease [35]. Notably, LPCs only assist with liver regeneration when hepatocyte regeneration is impaired, a common occurrence in human CLD [92, 93]. However, the overall contribution of LPCs to liver regeneration is still debated [93, 94]. Regardless, LPCs can contribute to liver regeneration during chronic liver injury; therefore, understanding how hAEC therapy impacts their biology is warranted.

It is difficult to discern the exact impact hAEC therapy will have on LPCs. On the one hand, several of the factors such as IGF2 and galectin 3 which are secreted by hAECs may stimulate LPC proliferation [95–97]. Conversely, the anti-inflammatory effects of hAECs would indirectly suppress the LPC response as MDMs and their associated inflammatory factors are important stimulators of LPC activity [98–100]. Additionally, LPC expansion is closely associated with the progression of liver fibrosis; hence, the antifibrotic effects of hAECs would likely dampen the LPC response [101, 102]. Consequently, hAEC-based therapies have the potential to both promote and inhibit liver regeneration by LPCs. To this end, characterisation of the effects hAECs have on LPC behaviour is warranted to fully understand the mechanism underpinning hAEC therapy.

## 8. Conclusion

Preclinical research over the last decade has illustrated the potential of hAEC-based therapies as a treatment for CLD (Table 1). These studies consistently show that the infusion of hAECs or their secretome reduces hepatic inflammation and fibrosis by modulating the activity of HSCs, macrophages, and other inflammatory cells. However, there are still many unanswered questions regarding the mechanisms behind these effects. Nevertheless, hAECs have progressed to clinical trials. A phase I pilot study aimed at evaluating the safety of intravenously administered hAECs in patients with end-stage CLD is currently underway [103]. Furthermore, outcomes from a phase I trial of allogeneic hAEC therapy in preterm infants with established bronchopulmonary dysplasia demonstrated no adverse effects, suggesting that hAECs will be safe to use in patients with CLD [104]. Looking ahead, research should aim to identify the factors secreted by hAECs that exert their beneficial effects. This will assist in designing future clinical trials and may lead to the development of an antifibrotic therapy based on the hAEC secretome. This cell-free approach could potentially be simpler, safer, and more commercially viable than whole-cell therapy.

## Figures and Tables

**Table 1:** Summary of results from studies using hAEC-based therapies in models of liver disease.

Study	Injury model	hAEC treatment	Main results
Manuelpillai et al. [22]	C57BL/6 mice administered CCl_4_ twice weekly for 4 weeks	Intraperitoneal injection of whole hAECs	Decreased liver injury, inflammation, and fibrosis
Sant'Anna et al. [105]	Bile duct ligation in Wistar rats for 6 weeks	Amniotic membrane place over ligation site	Reduced liver fibrosis
Manuelpillai et al. [36]	C57BL/6 mice administered CCl_4_ twice weekly for 12 weeks	Single and double dose of whole hAECs by intraperitoneal injection	Decrease fibrosis, decreased macrophage infiltration, increased M2 polarisation, and reduced T-cell infiltration
Ricci et al. [106]	Bile duct ligation in Sprague Dawley rats for 6 weeks	Fresh or cryopreserved amniotic membrane place over ligation site	Fresh and cryopreserved amniotic membrane produced the same antifibrotic effects
Alhomrani et al. [24]	C57BL/6 mice administered CCl_4_ twice weekly for 12 weeks	Tail vein injection of hAEC-CM or hAEC-derived exosomes	hAEC-CM and exosomes derived from hAECs reduce liver inflammation and fibrosis
Kuk et al. [45]	C57BL/6J mice on a Western fast food diet for 42 weeks	Multiple intraperitoneal injections of either whole hAECs or hAEC-CM	hAEC-CM reduces inflammation and fibrosis

CCl_4_: carbon tetrachloride; hAEC: human amnion epithelial cell; hAEC-CM: human amnion epithelial cell-conditioned medium.
